# The underlying cause of the simple virilizing phenotype in patients with 21-hydroxylase deficiency harboring P31L variant

**DOI:** 10.3389/fendo.2022.1015773

**Published:** 2023-02-14

**Authors:** Zhiyuan Zhao, Yinjie Gao, Lin Lu, Anli Tong, Shi Chen, Wei Zhang, Xiaoxia Zhang, Bang Sun, Xueyan Wu, Jiangfeng Mao, Xi Wang, Min Nie

**Affiliations:** ^1^ Department of Endocrinology, National Health Commission (NHC) Key Laboratory of Endocrinology (Peking Union Medical College Hospital), Peking Union Medical College Hospital, Peking Union Medical College, Chinese Academy of Medical Sciences, Beijing, China; ^2^ State Key Laboratory of Complex, Severe and Rare Diseases, Peking Union Medical College Hospital, Peking Union Medical College, Chinese Academy of Medical Sciences, Beijing, China

**Keywords:** classical simple virilizing, promoter variation, 21- hydroxylase deficiency, P31L, congenital adrenal hyperplasia

## Abstract

**Objective:**

To analyze the relationship between genotype and phenotype in 21-Hydroxylase deficiency patients harboring P31L variant and the underlying mechanism.

**Methods:**

A total of 29 Chinese patients with 21-OHD harboring P31L variant were recruited, and the detailed clinical features of the patients were extracted and analyzed retrospectively. The TA clone combined with sequencing of the region containing the promotor and exon1 of *CYP21A2* was performed to determine whether the variants in promotor and P31L aligned in cis. We further compared the clinical characteristics of 21-OHD patients between the promoter variant group and no promoter variant group.

**Results:**

Among the 29 patients diagnosed with 21-OHD harboring P31L variant, the incidence of classical simple virilizing form was 62.1%. Thirteen patients owned promoter variants (1 homozygote and 12 heterozygote) and all exhibited SV form. The promoter variants and the P31L variant were located in the same mutant allele as validated by TA cloning and sequencing. There were statistically significant differences in clinical phenotype and 17-OHP level between the patients with and without promoter region variations (*P*<0.05).

**Conclusion:**

There exists high incidence (57.4%) of SV form among the 21-OHD patients harboring P31L variant, and the underlying mechanism is partially due to both the promoter variants and P31L aligning in cis on one allele. Further sequencing of promoter region will provide important hints for the explanation of phenotype in patients harboring P31L.

## Introduction

21-hydroxylase deficiency (21-OHD), ranks among one of the most frequent inborn errors of the adrenal endocrine metabolism following an autosomal recessive trait (1). It is characterized by the impairment of cortisol synthesis with or without aldosterone deficiency, and increased androgen synthesis (1, 2). Based on the clinical manifestations, 21-OHD can be classified into three types: the classical salt-wasting (SW) form (approximately 75% of the classic 21-OHD), classical simple virilizing (SV) form (about 25% of the classic 21-OHD) and nonclassical form (NC-21OHD). The SW form is characterized by life-threatening adrenal crises in the neonatal period accompanied with hyperandrogenemia causing sexual ambiguity in affected females (3). The classical SV form usually exhibited precocious puberty combining with accelerated linear growth velocity, the affected females present virilization of external genitalia (i.e., clitoromegaly) or urogenital sinus in the early postnatal period (4). The NC-21OHD may be asymptomatic or clinically mild in the early stages and tend to display signs or symptoms of androgen excess until preadolescent, adolescent or young adult period, characterized by hirsutism, acne, menstrual disorders, subfertility and recurrent miscarriage (5–8).

The original cause of 21-OHD can be ascribed to the decrease or abrogation of P450C21 enzyme activity, which is encoded by the *CYP21A2* gene. *CYP21A2* is located on chromosome 6p21.3 adjacent to a nonfunctional pseudogene *CYP21A1P*. The *CYP21A2* and *CYP21A1P* genes show a high homology, with a nucleotide identity of 98% in their exon and 96% in their intron sequences (9–11). Approximately 95% of *CYP21A2* pathogenic variants are *CYP21A1P*-derived or large deletions due to non-homologous recombination events in meiotic (3, 12–14).

The clinical phenotype of 21-OHD is usually well correlated with the residual enzyme activity of mutant P450C21 resulted from *CYP21A2* variant (1, 15). Variants leading to 0 to 1% enzymatic activity remaining of mutant P450C21 typically correspond to classical SW 21-OHD, such as 30KB deletions, L308Ffs*6(F308+T), R357W, E6 Cluster (I237D/V238E/M240K), 8bp deletion(E3Δ8bp, c.332_339del GAGACTAC, p.G111Vfs∗21), Q319* and c.293-13A/C>G(i2g). Variants resulting in nearly 1% to 2% enzyme activity retaining of mutant P450C21 frequently cause the classical SV 21-OHD, as demonstrated by I173N. Mutant P450C21 preserving 20% to 60% enzyme activity (e.g.,P31L, V282L and P454S) usually bring about the NC-21OHD (16). About 65–70% of patients with 21-OHD are compound heterozygous, while the clinical phenotype is generally considered to be determined by the less severely affected allele (4, 14).

However, some discrepancies between genotype/phenotype correlation had been found in 21-OHD patients. For example, P31L mutant P450C21 usually lead to NC-21OHD phenotype due to its retention of more than 50% residual enzyme activity (17), while the patients with P31L variant might present SV phenotype (8, 18). Some investigators showed that it could be the result of the occurrence of both promoter region variants and P31L in the same allele (19). In this study, we mainly focused on the relationship between genotype and phenotype in 21-OHD patients harboring P31L variant and its mechanism to augment our understanding of 21-OHD.

## Subjects and methods

### Subjects

A total of 29 Chinese patients with 21-OHD harboring P31L variant identified by genetic testing were recruited for this study, who presented to Peking Union Medical College Hospital (Beijing) between 2003 and 2021.

The study was approved by the ethics committee of Peking Union Medical College Hospital (No.JS-2111).

### Study design

This was a retrospective study. Detailed medical data pertaining to age, sex, previous medical history (clinical diagnosis and history of vulvar surgery), clinical presentations (hirsutism, acne, menstrual abnormalities, clitoromegaly or labial fusion and precocious pubarche), laboratory data including cortisol(F), testosterone(T), 17α-hydroxyprogesterone (17- OHP) and plasma adrenocorticotropic hormone (ACTH) at baseline (without treatment) or after discontinuation of treatment were extracted and analyzed. The variants of *CYP21A2* were identified.

### Laboratory test

Plasma adrenocorticotropic hormone (ACTH) and serum cortisol(F) at 8:00 AM were measured by chemiluminescence immunoassay (Advia Centaur XP, Bayer). Serum testosterone (T) was measured with chemiluminescence (ACS:180; Automatic Chemiluminescence Systems, Bayer). 17α-hydroxyprogesterone (17-OHP) concentrations was determined by radioimmunoassay (Active 17α-OHP Progesterone DSL-5000, DSL). The intra and inter assay coefficients of variation were 5.6% and 6.6% for T, 6.7% and 8.2% for ACTH,5.3% and 5.7% for serum cortisol, 3.9% and 5.6% for 17-OHP, respectively.

### Variant analysis of the *CYP21A2* gene

Genomic deoxyribonucleic acid (DNA) from the peripheral blood leukocytes were obtained from all patients using a standard procedure (Omega Blood DNA Midi Kit, Omega Bio-Tek, USA). Multiplex Ligation-dependent probe amplification (MLPA) and PCR combined with sequencing were employed to detect the variants in the region between 700 bp upstream from the start codon ATG of *CYP21A2* gene and the entire *CYP21A2*. The specific primer sequences and PCR amplification methods were described previously (20). The sequencing results were compared with the reference sequence NM_000500.9 of the *CYP21A2* through the NCBI website to determine the variants. MLPA (P050-C1 CAH Kit, MRC Holland) was performed according to the manufacturer’s instructions.

### TA cloning

The subjects harboring variants both in promoter region and P31L were further sequenced. The fragments containing 5’ UTR and exon1 of *CYP21A2* were cloned into pMD19T vector by TA cloning kit (Takara, Bio, Inc.) according to the instruction to determine whether the variants in promoter region were aligned with P31L in cis.

### Statistical analysis

All statistical analyses were performed using SPSS (version 26, SPSS Inc., IBM). Shapiro-Wilk test was performed to determine whether the continuous variables conform to normal distribution. The normally distributed quantitative variables were represented as mean ± standard deviation ( $\bar{x}$ ± s), and the non-normally quantitative distributed variables were expressed as median (upper and lower quartiles) [M (Q1, Q3)]. Comparison between two groups was performed using independent t-test or Mann–Whitney U test, as appropriate. Categorical variables were expressed as cases (n) and percentages and were compared by the Chi-square test or Fisher exact test. *P* value less than 0.05 was considered statistically significant.

## Results

### Clinical characteristics of 21-OHD patients harboring P31L

Among the 29 patients diagnosed with 21-OHD harboring P31L variant, 18 patients presented classical SV form, and the remaining 11 patients manifested as nonclassical form. The patients in the SV group were younger than NC group (18.72 ± 6.96 years v.s. 25.55 ± 6.49years, respectively) and with higher serum 17-OHP (58.95 ± 26.20ng/ml v.s. 19.91 ± 16.94ng/ml, respectively). No significant differences were found in other laboratory results (**Table 1**).

**Table 1:** Comparison of clinical characteristics of 21-OHD patients harboring P31L between SV and NC group.

Clinical characteristics	SV (n=18)	NC (n=11)	*P*
Age	18.72 ± 6.96	25.55 ± 6.49	0.014
17-OHP	58.95 ± 26.20	19.91 ± 16.94	<0.001
T	2.15 (0.98,3.51)	1.79 (1.27,2.34)	0.637
F	10.22 (5.93,14,64)	10.04 (13.07,17.08)	0.239
ACTH	159.40 (58.80,233.00)	87.20 (49.00,177.00)	0.384

SV, simple virilizing form; NC, non classical form.

17-OHP, 17-Hydroxyprogesterone; ACTH, Adrenocorticotropic hormone; T, Testosterone; F, cortisol.

Normal reference interval:17-OHP: 0.31-2.17(M); 0.10-0.80ng/ml(F); T: 1.75-7.81ng/ml(M), 0.10-0.75ng/ml(F); ACTH: 0-46pg/ml, F:4-22.3ug/dl.

### Variants in the promoter of *CYP21A2* in the 21-OHD patients harboring P31L

A total of 13 patients have the variants in promoter region, and 12 patients were compound heterozygote, while one patient was homozygote (**Table 2**). TA cloning and sequencing showed that the promoter variants and the P31L variant were located in the same allele, and the detailed promoter variants are listed as follows: -4C→T,-103A→G,-110T→C,-113G→A,-126C→T,-198InsT,-201C→T,-212T→C,-284T→G,-286A→G,-297A→C,-298T→C,-310G→C,-448InsA. The above variants are consistent with the corresponding locus in the *CYP21A1P* gene (**Supplementary Figure S1**).

**Table 2:** Genotypes and phenotypes of 29 patients with 21-OHD harboring P31L.

Patients No.	Clinical category	Clinical characteristics	Allele 1	Allele 2
21OHD01	SV	H,M,C	promoter variant, P31L	del
21OHD02	SV	C, CP	promoter variant, P31L	exon1-3 del
21OHD03	SV	H, M, C, A, CP	promoter variant, P31L	Del
21OHD04	SV	H, C, CP	promoter variant, P31L	E3Δ8bp
21OHD05	SV	H, M, C, A	promoter variant, P31L	promoter variant, P31L
21OHD06	SV	C	promoter variant, P31L	I173N
21OHD07	SV	H, C, PP, CP	promoter variant, P31L	I173N
21OHD08	SV	H, M, C, A, CP	promoter variant, P31L	Q319X
21OHD09	SV	C, CP	promoter variant, P31L	I173N
21OHD10	SV	H, M, C, A	promoter variant, P31L	i2g
21OHD11	SV	H, C, CP	promoter variant, P31L	i2g
21OHD12	SV	C, CP	promoter variant, P31L	I173N
21OHD13	SV	H, M, A, CP	promoter variant, P31L	I173N
21OHD14	SV	H, C, PP, CP	P31L	exon4-8 deletion
21OHD15	SV	C	P31L	Del
21OHD16	NC	H, M	P31L	I173N
21OHD17	NC	H, M	P31L	Del
21OHD18	NC	H, M, A	P31L	Del
21OHD19	NC	H, M, A	P31L	i2g
21OHD20	NC	H, M, A	P31L	Del
21OHD21	NC	H, M	P31L	I173N
21OHD22	NC	H, M	P31L	I173N
21OHD23	NC	H, M, A	P31L	I173N
21OHD24	NC	H, M, A	P31L	V282L
21OHD25	NC	H, M, A	P31L	i2g
21OHD26	SV	H, M, C, A	P31L	Del
21OHD27	SV	H, M, C	P31L	I173N
21OHD28	NC	H, M	P31L	I173N
21OHD29	SV	H, M, C	P31L	P31L

SV, the simple virilizing form; H, hirsutism; M, menstrual abnormalities; C, clitoromegaly or labial fusion; A, acne; PP, precocious pubarche; CP, clitoroplasty.

Promoter variants:-4C→T,-103A→G,-110T→C,-113G→A, -126C→T, -198InsT,-201C→T, -212T→C,-284T→G,-286A→G,-297A→C,-298T→C,-310G→C,C.-448InsA.

### Comparison of clinical characteristics of 21-OHD patients between promoter variant group and no promoter variant group

The 29 patients were divided into promoter variant (PV) group and no promoter variant(NPV) group according to whether the promoter variations exist or not.17-OHP level were higher in the PV group than the NPV group. The clinical phenotype of 21-OHD patients in the PV group were quite different from the NPV group (*P*<0.05). All the 13 patients in the PV group were SV form, and 9 of them had undergone clitoroplasty. While most (11/16,68.8%) 21-OHD patients in the NPV group showed NC-21OHD form and the 5 other patients SV form. No statistical difference was noted in the other laboratory results and residual enzyme activity of the variant on the other allele (other than P31L located allele) (**Table 3**).

**Table 3:** Comparison of clinical characteristics of 21-OHD patients with both promoter variation and P31L variation versus P31L variation alone.

Clinical characteristics		PV (n=13)	NPV (n=16)	*P*
Age		23.20 ± 5.81	25.92 ± 5.55	0.570
17OHP		64.63 (52.99,72.86)	18.71 (8.72,53.35)	0.014
T		1.01 (0.85,2.74)	1.99 (1.40,3.37)	0.079
F		10.11 ± 3.06	13.87 ± 4.85	0.244
ACTH		177.00 (48.23,240.00)	101.60 (56.63,175.75)	0.865
Cases of patients undergoing clitoroplasty(%)		9 (69.23%)	1 (6.25%)	0.001
clinical phenotype	NC	0	11	<0.001
	SV	13	5	
residual enzyme activity of the mutation on the other alelle (other than P31L)	0-1%	7	8	1.000
	1%-2%	5	6	
	20%-60%	1	2	

PV, promoter variant group; NPV, no promoter variant group.

SV, simple virilizing form; NC, non classical form; 17-OHP, 17-Hydroxyprogesterone; ACTH, Adrenocorticotropic hormone; T, Testosterone; F, cortisol.

Normal reference interval:17-OHP: 0.31-2.17(M);0.10-0.80ng/ml(F);T:1.75-7.81ng/ml(M), 0.10-0.75ng/ml(F);ACTH: 0-46pg/ml, F:4-22.3ug/dl.

## Discussion

This study investigated the relation of genotype and phenotype and the occurrence of promoter variants among 21-OHD patients harboring P31L variant in a single medical center. Our findings demonstrated that the incidence of SV was 57.4% in 21-OHD patients harboring P31L variant, of which 84.6% patients were caused by promoter variants besides P31L on the same allele of *CYP21A2*.

In recessive disorders, the clinical phenotype of 21-OHD is usually determined by the activity of the milder variant in compound heterozygotes. According to the *in vitro* studies, residual enzyme activity of the P31L mutant usually leads to a relatively mild NC-21OHD (21, 22). In this study, the ratio of the classical SV to the NC-21OHD female patients harboring P31L variants was 18:11. Another study from Argentina found that the ratio of the SV form to the NC-21OHD patients with P31L variants was 1:1 (23). Other studies have found that in different 21-OHD patient series harboring P31L variants, the incidence of SV clinical phenotype ranged from 20% to 100% (4, 22, 24–29). As described in previous literature (30), comparing with other variants causing NC-21OHD, the patients affected by P31L tended to have more severe clinical phenotype and even exhibit the SV form. Our study showed the similar results, which strongly indicates that there also existed the genotype/phenotype discrepancies among the Chinese 21-OHD patients harboring P31L. For 21-OHD patients harboring P31L variants, additional attention should be paid to whether more severe clinical phenotype exists or not, so as to provide important basis for precise clinical intervention.

In this study, we found that all the 21-OHD patients with promoter variants in cis with the P31L variant presented SV phenotype. These results suggest that, in addition to the impairment of P450C21 protein activity by the P31L variant, promoter variants may further affect the function of the enzyme, and some evidence supported it. It was reported that c.-113G>A variant of *CYP21A2* could reduce the basal transcriptional activity to 20% of *CYP21A2 (* 31), and the c.-126C>T could decrease the transcriptional activity of *CYP21A2* to 52% (32). The transcriptional activity of variants c.-126C > T, c.-113G > A, c.-110T > C and c.-103T > C in the promoter is reduced to 20% of the wild type and correlated with the SV 21-OHD (20, 33, 34). Our TA clone sequencing results demonstrated that *CYP21A2* promoter variants within c.-500bp to c.-1bp upstream of the ATG occurred in all thirteen patients, including c.-448InsA, c.-310G>C, c.-298T>C, c.-297A>C, c.-286A>G, c.-284T>G, c.-212T>C, c.-201C>T, c.-198InsT, c.-126C>T, c.-113G>A, c.-110T>C, c.-103A>G, c.-4C>T, along with c.91C>T (P31L), are consistent with the corresponding locus of *CYP21A1P*, implying that the gene conversion from the *CYP21A1P* to the *CYP21A2*. For 21-OHD patients tested out P31L variant, further sequencing of genetic locus in the promoter region are needed to obtain the comprehensive and complete molecular diagnosis results, thus providing important basis for subsequent precise clinical treatment and reliable genetic risk assessment.

In our study, the promoter region variation might account for 72.2% of all the classical SV form 21-OHD patients harboring P31L, which is similar to the previous results (19, 23, 34) (**Table 4**), and their general occurrence of promoter variant in SV 21-OHD patients harboring P31L were 84.6%. Our study demonstrated that 5 patients in the NPV group also showed the clinical phenotype of SV form. This implied that other reasons (besides promoter variants) could account for the more severe clinical phenotype of 21-OHD patients haboring P31L. The following three mechanisms were reported. First, *CYP3A7* gene and its transcriptional regulator constitutive androstane receptor (CAR) might be involved in fetal virilization in female 21-OHD. Specifically, the CAR variant could account for a higher degree of external genitalia virilization (35). Second, CAG repetition in the exon 1 of androgen receptor gene matters and SV 21-OHD patients tended to have fewer CAG repeats (36). Third, the alternative pathway of androgen biosynthesis during embryonic development and corresponding proteins (SRD5A1, AKR1C1/3, HSD17B6, etc.) may be involved in fetal virilization in females, through which the 17-OHP accumulated in the 21-OHD may be converted to the androgen and thus aggravate female virilization (37).

**Table 4:** The summary of incidence of SV phenotype and promoter variants in 21-OHD patients harboring P31L.

The number of patients	SV incidence (No.)	Detection rate* (No.)	Reference
15	40% (6)	100% (6)	(23)
15	80% (12)	100% (12)	(34)
9	33.3% (3)	66.7% (2)	(19)
29	62.1% (18)	72.2% (13)	Present study
Total (68) #	57.4%(39)	84.6% (33)	–

*Detection rate of promoter region in SV patients.

# The total number of 21-OHD patients harboring P31L undergoing sequence of promoter region.

## Limitations

There are some limitations to this research. This was a retrospective study, hence, the medical information, including the neonatal virilization of external genitalia, might have been affected by recall bias. This might have resulted in an underestimation of the incidence of SV form among the 21-OHD patients harboring P31L variant.

## Conclusion

There exists high incidence (57.4%) of SV form among the 21-OHD patients harboring P31L variant, and the underlying mechanism is partially due to both the promoter variants and P31L aligning in cis on one allele. Further sequencing of promoter region will provide important hints for the explanation of phenotype in patients harboring P31L.

## Data availability statement

The original contributions presented in the study are included in the article/supplementary files, further inquiries can be directed to the corresponding author/s.

## Ethics statement

The studies involving human participants were reviewed and approved by ethics committee of Peking Union Medical College Hospital (No.JS-2111). The patients/participants provided their written informed consent to participate in this study. Written informed consent was obtained from the individual(s) for the publication of any potentially identifiable images or data included in this article.

## Author contributions

MN conceived the project, designed the experiments. LL, AT, SC, XiW, JM and XuW collected the clinical data, YG, WZ, BS and ZZ performed the experiments. MN and ZZ analyzed the data and wrote the manuscript. All authors contributed to the article and approved the submitted version.
