# Supplementary material for: The underlying cause of the simple virilizing phenotype in patients with 21-hydroxylase deficiency harboring P31L variant
Source: Front Endocrinol (Lausanne). 2023 Feb 14;13:1015773. doi: 10.3389/fendo.2022.1015773 (PMC9972294; doi:10.3389/fendo.2022.1015773)
Supplement: Supplementary file 1 [file Table_1.docx]

**Supplementary**

**Table S1. PCR primers and the amplification methods**

a. PCR and sequencing primers for *CYP21A2* gene

| Primer name | Primers | Sequences (5‘–3’) | Tm(℃) | Product size (bp) |
| --- | --- | --- | --- | --- |
| CYP21A2710A | F | CCACAACATGCGAACAATAC | 58.6 | 1427 |
|  | R | GGGAGTAGTCTCCCAAGGAC |  |  |
| CYP21A2B1B2 | F | TTTTGTTCTTCAGGCGATTCA | 58.1 | 1155 |
|  | R | TCCAGAGCAGGGAGTAGTCTC |  |  |
| CYP21A2C1C2 | F | CCGGACCTGTCCTTGGGAGACTACT | 63.2 | 2568 |
|  | R | CTGAGTGGCTGGGTGAAATGGAACA |  |  |
| CYP21A2P1 | F | TCGGTGGGAGGGTACCTGAAG | 59.3 | 1514 |
|  | R | GCATCTCCACGATGTGA |  |  |

F: forward prime, R: [reverse](javascript:;) primer

b. Amplification methods

①PCR reaction system (30ul) was as follows: DNA template 3ul (100ng), upstream and downstream primers 1.5ul (10umol/l) and 2 x GoldStar MasterMix 15ul, with the remaining volume filled with water. 2 x GoldStar MasterMix is a premixed system consisting of GoldStar DNA Polymerase, PCR buffer, Mg2+, dNTPs and PCR stabilizers and enhancers (TransGen Biotech, Beijing).

②The condition of PCR amplification was as follows: predenaturation at 95℃ for 5 minutes, denaturation at 95℃ for 30 seconds, annealing at 58–63℃for 30 seconds, and extension at 72℃ for 3 minutes. A total of 35 cycles were carried out, with final extension at 72℃ for 10 minutes.

**FigureS1. TA clone sequencing results of 21-OHD patients with heterozygous P31L variation in cis with the promoter region variation**


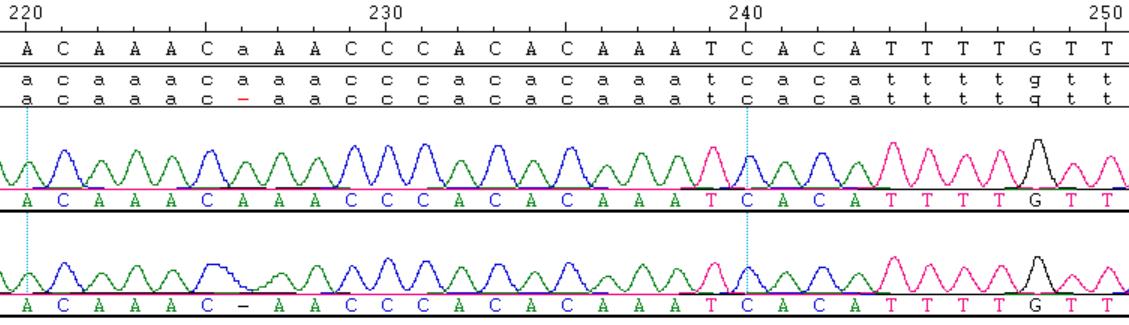
  **C.-448InsA**

**Allele1**

**Allele2**

***CYP21A1P***

***CYP21A2***


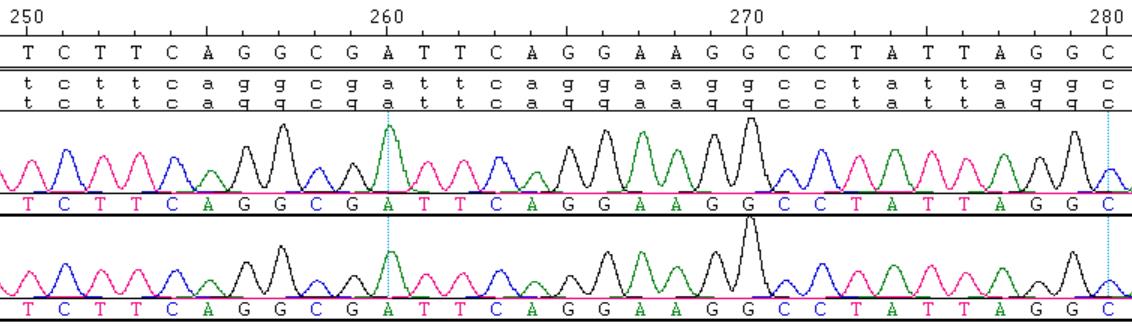


***CYP21A1P***

***CYP21A2***

**Allele1**

**Allele2**


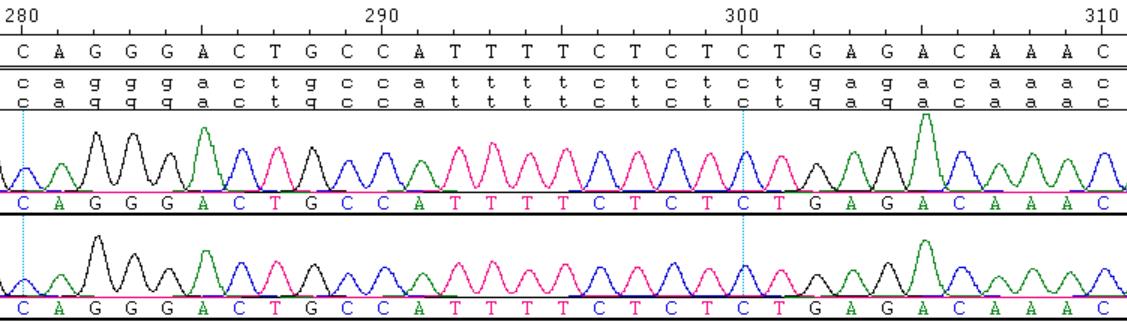


**Allele1**

**Allele2**

***CYP21A1P***

***CYP21A2***

***CYP21A1P***

***CYP21A2***

**Allele1**

**Allele2**


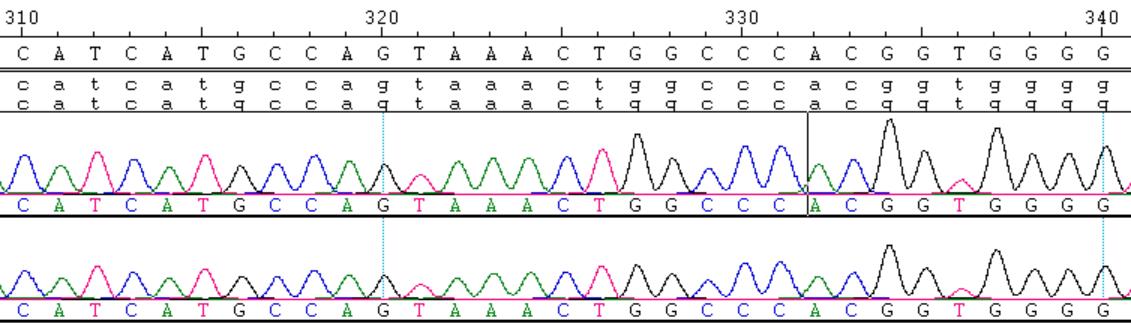


**C.-310G/C**


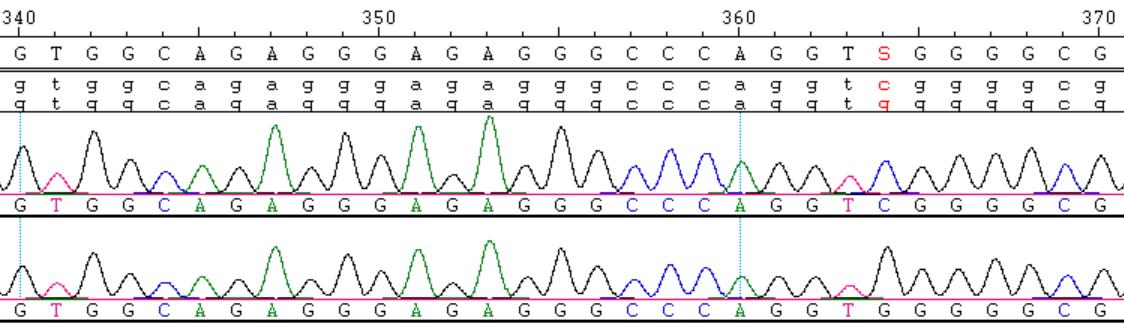


**Allele1**

**Allele2**

***CYP21A1P***

***CYP21A2***

**C.-298T/C C.-297A/C C.-286A/G C.-284T/G**


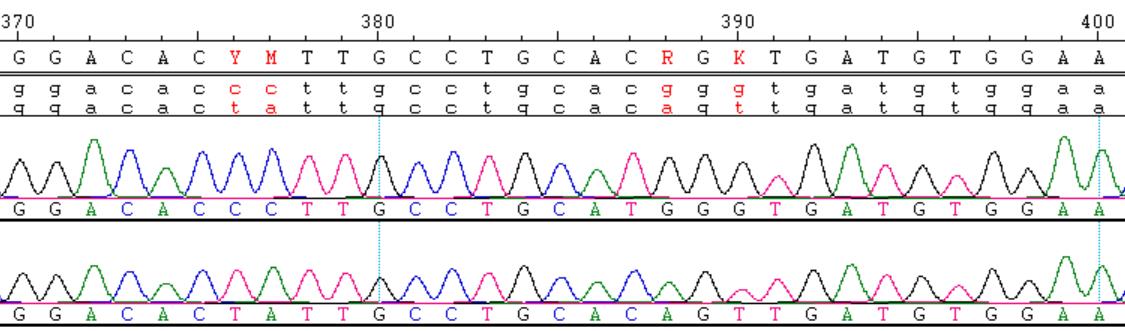


***CYP21A1P***

***CYP21A2***

**Allele1**

**Allele2**


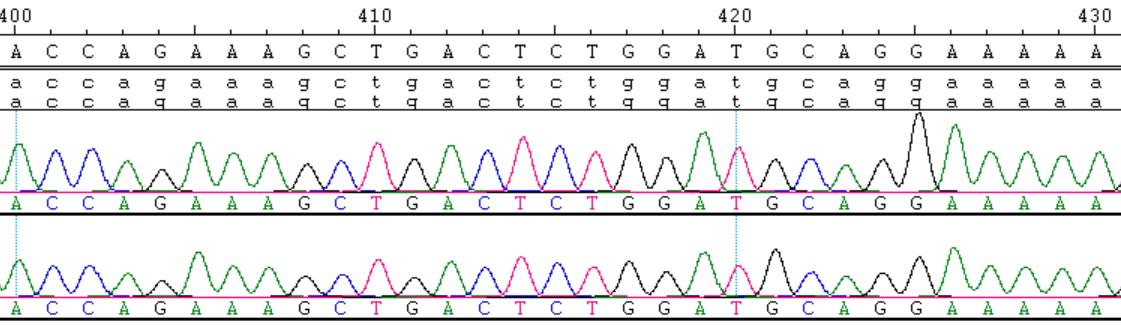


**Allele1**

**Allele2**

***CYP21A1P***

***CYP21A2***


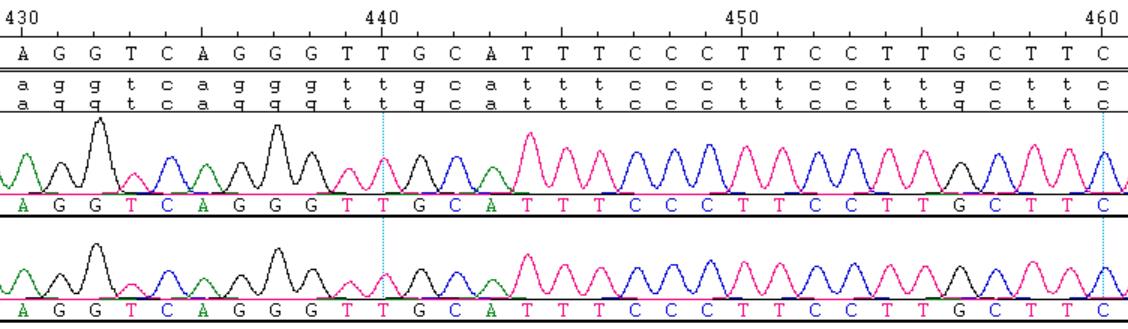


***CYP21A1P***

***CYP21A2***

**Allele1**

**Allele2**

**C.-212T/C -201C/T -198insT**


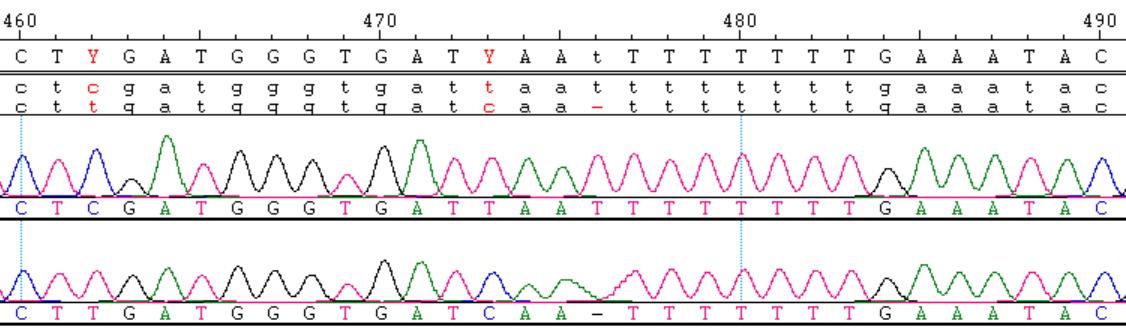


**Allele1**

**Allele2**

***CYP21A1P***

***CYP21A2***


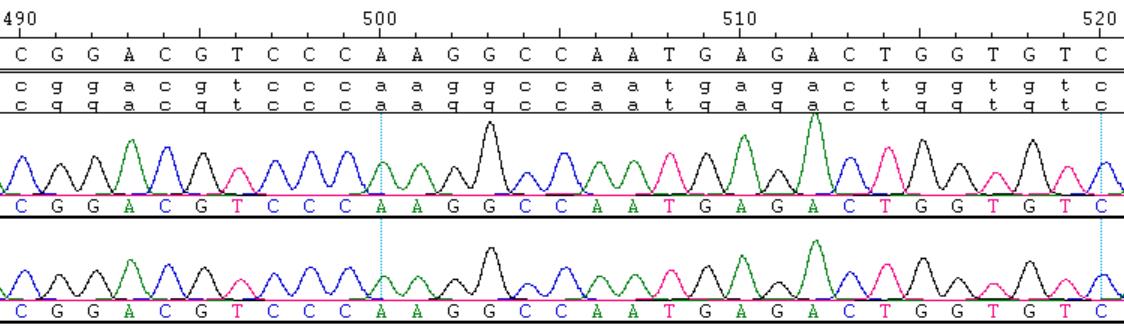


**Allele1**

**Allele2**

***CYP21A1P***

***CYP21A2***

**C.-126C/T**


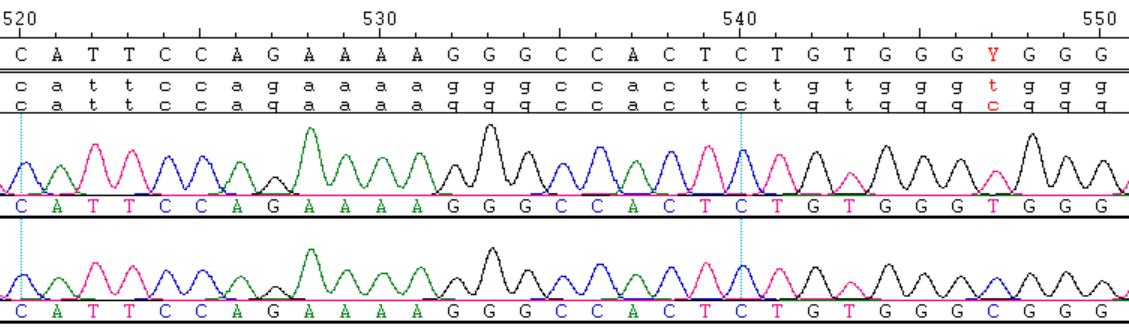


***CYP21A1P***

***CYP21A2***

**Allele1**

**Allele2**

**C.-113G/A C.-110T/C C.-103A/G**


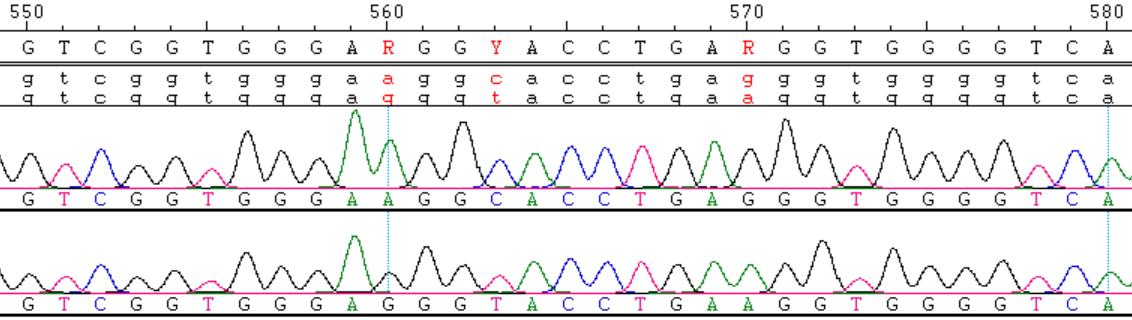


**Allele1**

**Allele2**

***CYP21A1P***

***CYP21A2***


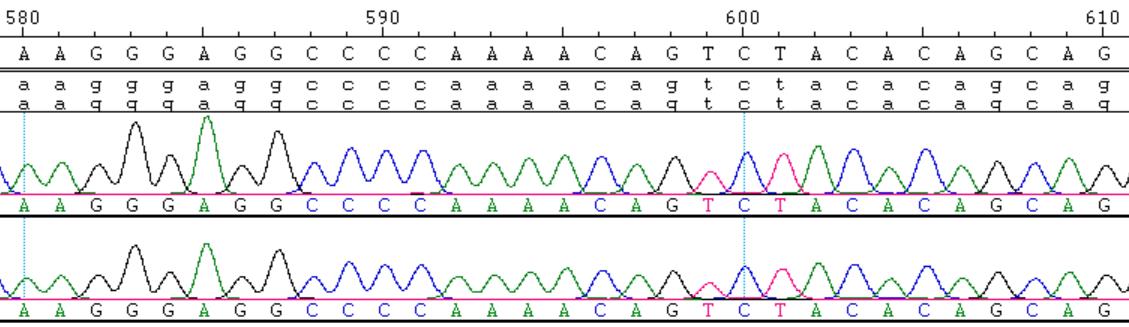


***CYP21A1P***

***CYP21A2***

**Allele1**

**Allele2**

**Allele1**

**Allele2**

***CYP21A1P***

***CYP21A2***


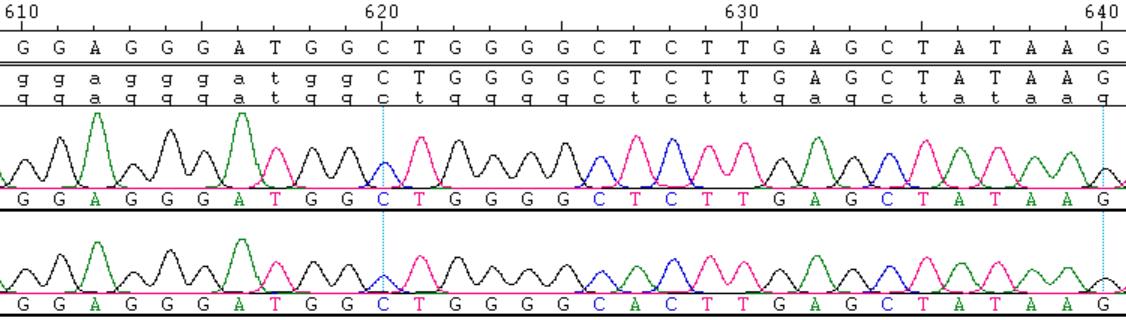


**C.-4C/T**


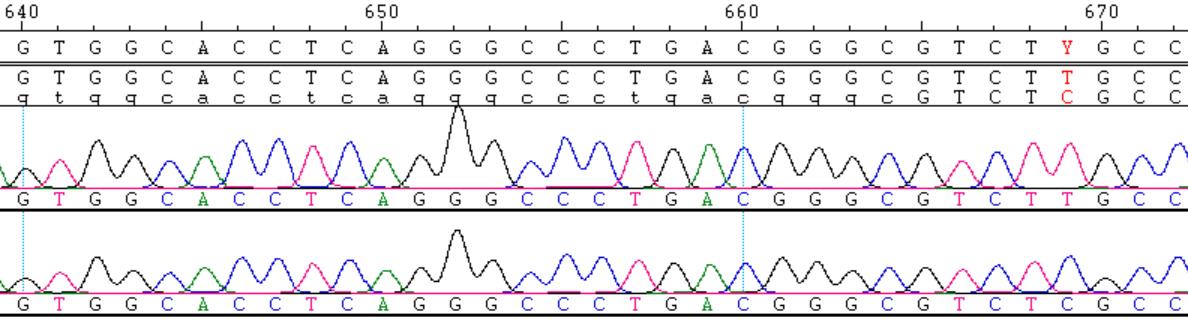


**Allele1**

**Allele2**

***CYP21A1P***

***CYP21A2***


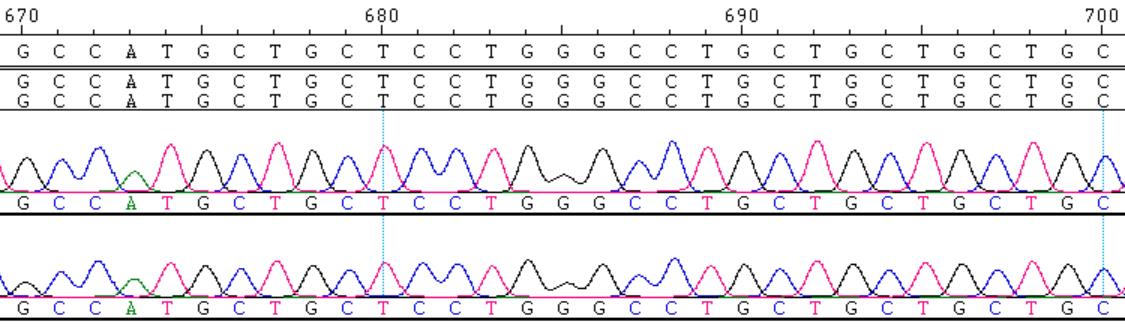


**Allele1**

**Allele2**

***CYP21A1P***

***CYP21A2***


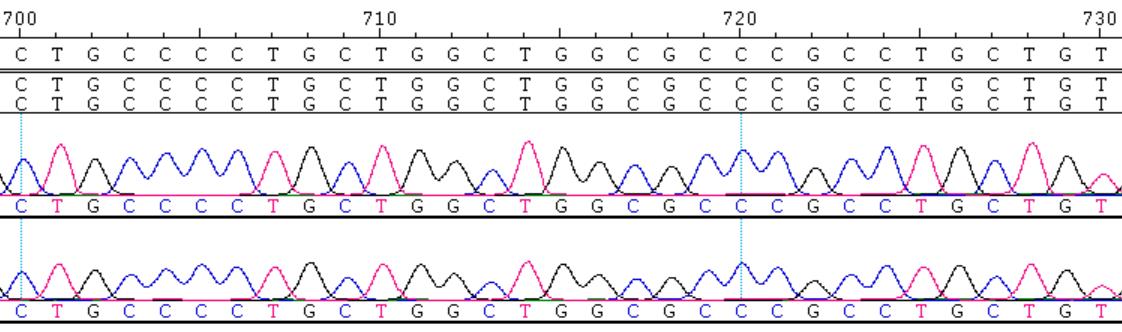


**Allele1**

**Allele2**

***CYP21A1P***

***CYP21A2***

**C.91C/T**


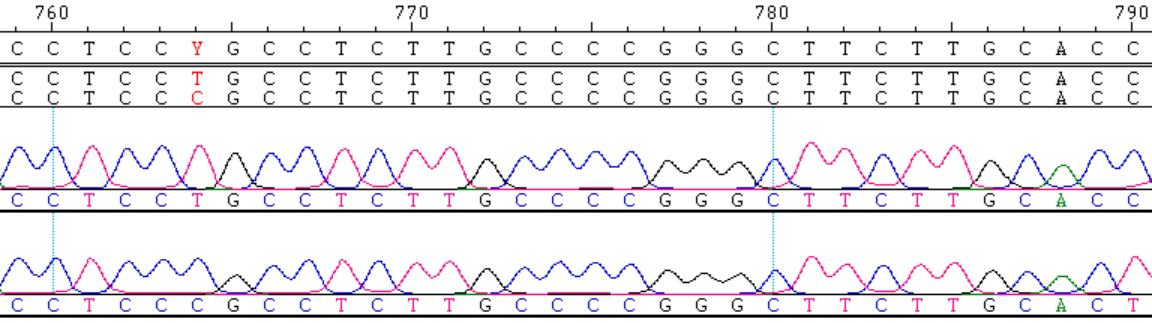


**Allele1**

**Allele2**

***CYP21A1P***

***CYP21A2***
